# A Multicenter Exploration of Sick Building Syndrome Symptoms in Malaysian Schools: Indoor Pollutants, Microbial Taxa, and Metabolites

**DOI:** 10.3390/metabo15020111

**Published:** 2025-02-10

**Authors:** Yi Zhang, Yongqi Bu, Yang Chen, Peian Chen, Bingqian Du, Jamal Hisham Hashim, Zailina Hashim, Gunilla Wieslander, Dan Norbäck, Yun Xia, Xi Fu

**Affiliations:** 1 NMPA Key Laboratory for Technology Research and Evaluation of Pharmacovigilance, Guangdong Provincial Engineering Research Center of Public Health Detection and Assessment, School of Public Health, Guangdong Pharmaceutical University, Guangzhou 510006, China; 2 Guangdong-Hong Kong-Macao Joint Laboratory for Contaminants Exposure and Health, Guangzhou 510006, China; 3 State Key Laboratory of Swine and Poultry Breeding Industry, South China Agricultural University, Guangzhou 510642, China; 4 Department of Environmental Health & Occupational Safety, Faculty of Health Sciences, Universiti Selangor, Shah Alam 40000, Selangor, Malaysia; 5 Department of Environmental and Occupational Health, Faculty of Medicine and Health Sciences, Universiti Putra Malaysia, Serdang 43400, Selangor, Malaysia; 6 Occupational and Environmental Medicine, Department of Medical Science, University Hospital, Uppsala University, 75237 Uppsala, Sweden

**Keywords:** indoor NO_2_, multiomics, mediation, synthetic chemicals

## Abstract

Background: The role of the indoor microbiome in sick building syndrome (SBS) is well-recognized, yet prior studies have been limited to single-center analyses, limiting a broader understanding and applicability of their findings. Methods: We conducted a multicenter indoor microbiome and metabolome investigation for SBS, involving 1139 middle school students across three regions in Malaysia (Johor Bahru, Terengganu, and Penang). Using high-throughput amplicon sequencing and untargeted LC-MS, indoor microbiome and metabolites were characterized from classroom dust samples. Results: The study found that the prevalence of SBS symptoms was high across all three centers (51.0% to 54.6%). Environmental characteristics, including indoor NO_2_ and CO_2_ concentrations and total weight of indoor dust, were positively associated with SBS (*p* < 0.01, linear regression). *Curtobacterium* in Terengganu was negatively associated with SBS, and Clostridium perfringens in Johor Bahru was positively associated with SBS (*p* < 0.01, FDR < 0.05). Whereas all identified fungal taxa, including an uncharacterized *uc_f_Auriculariaceae*_sp., *Duportella kuehneroides*, and *Wallemia mellicola*, were positively associated with SBS (*p* < 0.01, FDR < 0.05) in Johor Bahru and Terengganu. Mediation analysis revealed that the adverse health effects of NO_2_ on SBS were partially mediated by the increased abundance of *uc_f_Auriculariaceae*_sp. (*p* < 0.05, total effect mediated 51.40%). Additionally, potential protective metabolites (S-adenosylmethionine, N-acetylserotonin, sphinganine, 4-hydroxy-2-quinolone, and (2E,4Z,8E)-Colneleic acid) were mainly derived from environmental microorganisms, conferring protective effects against nasal symptoms and tiredness. In contrast, synthetic chemicals were associated with higher SBS symptoms, inducing eye and nasal symptoms. Conclusions: This study emphasizes both the significance of fostering a balanced indoor microbiome/metabolite and the necessity to reduce exposure to deleterious substances, providing new insights for future targeted intervention strategies.

## 1. Introduction

Sick building syndrome (SBS) is a multifaceted phenomenon characterized by a constellation of non-specific symptoms that are typically associated with occupants’ time spent within a specific building, without being attributable to any definitive illness or cause [1]. The symptoms include but are not limited to headaches, dizziness, chronic fatigue, eye, nose or throat irritation, dry cough, dry or itchy skin, and difficulty in concentrating [2,3]. The prevalence of SBS can be high, posing significant health risks and burdens for indoor occupants. For example, studies reported that over 50% of bank officers in Nepal and hospital staff in Sivas experienced SBS symptoms [4,5].

Various factors influence the occurrence of SBS, including ventilation, chemical and biological contaminants (traffic pollutants, cleaning agents, pesticides, tobacco smoke, indoor mold, and mycotoxins), psychological factors (working stress, psychosocial environment, and job dissatisfaction), and physical factors (relative humidity, temperature, lack of natural light, and noise) [6,7,8,9,10]. The role of indoor microbial composition and diversity in SBS symptoms has started to be explored in recent years. Most studies have employed traditional cultivation methods and quantitative PCR [11], but two studies applied culture-independent high-throughput sequencing for microbiome profiling. One study found a protective/negative association between three microbial genera (*Rhodomicrobium*, *Scytonema*, and *Microcoleus*) and ocular and throat symptoms and fatigue [3]. Another study in China reported a higher prevalence of SBS symptoms in urban areas than in rural areas [2], and the abundance of microbial metabolic pathways related to the synthesis of B vitamins, gamma-aminobutyric acid (GABA), short-chain fatty acids (SCFAs), and peptidoglycan was negatively associated with SBS symptoms. These studies highlight the significant role of the indoor microbiome in SBS symptoms.

SBS is widely reported in office buildings and residential homes [9,10,12], but growing evidence indicates that SBS is also a common disease burden in schools, adversely affecting students and teachers [13,14,15,16,17]. For example, indoor air quality and dust have a significant impact on SBS in schools, with worse air quality correlating with a higher prevalence of SBS-related symptoms [15,16]. A study in female college dorms found a significant dose–response relationship between the use of air fresheners/perfumes and symptoms of fatigue and nasal irritation [15]. Another study reported that teachers’ work-related SBS symptoms were 2.9 times and 1.8 times higher in classrooms with highly toxic dust samples compared to those with non-toxic dust samples [17]. Investigating the school environment could provide new insights into protecting the health of staff and students.

Although there have been significant advancements in the field, there are several key limitations to SBS studies. First, previous indoor microbiome-SBS studies were restricted to the single center or a particular geographic location, making it unclear whether their findings can be generalized to other geographic locations [2,3]. Second, previous SBS studies have not applied untargeted metabolomics to profile environmental metabolites and chemicals [2,3]. Most studies have targeted specific chemicals, such as LPS or MVOCs [11], which do not capture the complexity of environmental chemical exposures for SBS. Only one study in Malaysia used an untargeted metabolomics approach and found a protective effect of pipecolic acid on nasal symptoms in school students [18]. The use of untargeted metabolite profiling methods in the study of environmental exposure and SBS will provide new insights into the health of indoor occupants.

This present study aims to address these gaps. We conducted a multicenter indoor microbiome and metabolome investigation, encompassing SBS prevalence among middle school students in multiple regions in Malaysia (Johor Bahru, Terengganu, Penang). LEfSe and three-level hierarchical linear regression models were applied to capture the key health associations of indoor microorganisms and metabolites. The impact of environmental characteristics on SBS-associated indoor microorganisms and SBS symptoms was investigated by mediation analysis. The study aims to shed light on potential prevention strategies to reduce the prevalence of SBS symptoms, thereby contributing to the improvement of indoor public health. The study also aims to improve the understanding of how environmental characteristics interact with indoor microbial components and contribute to the occurrence of SBS symptoms.

## 2. Materials and Methods

### 2.1. Study Design

**Figure 1:**
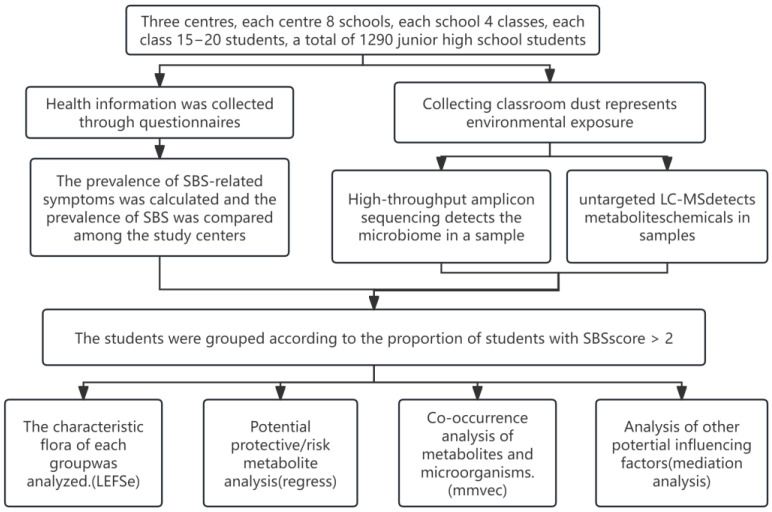
Research methodology flowchart.

This study was conducted across three centers in Malaysia: Johor Bahru, Terengganu, and Penang (Figure S1). In each center, eight junior high schools were randomly selected, and within each school, four classrooms were randomly selected. Questionnaires were randomly distributed to 15–20 students in each class. Dust samples were collected for microbial sequencing and profiling of metabolites and chemicals. The Ethics Committee and the National University of Malaysia Medical Research approved the study design and protocol, and all participants provided informed consent. Patients or the public were not involved in the design, conduct, reporting, or dissemination plans of this research. Our study design and protocol were approved by the Medical Research and Ethics Committee of the National University of Malaysia (Selangor, Malaysia) (UKM1.5.3.5/244/SPP/FF-345-2010, 3 November 2010). Informed consent was obtained from all participants. The research methodology flowchart is presented in Figure 1.

### 2.2. Questionnaire and Assessment of SBS Symptoms

Questionnaires were issued to the students, which contained questions about gender, age, smoking habits, and Sick Building Syndrome (SBS) symptoms. The SBS questions were adapted from the SBS questionnaire created by Uppsala University [19], which has been widely used in previous studies [2,9,20,21]. The SBS-related questions included “Do you have the following health concerns in the last three months?” and the symptoms contained six categories: (I) two questions on ocular symptoms—irritated eyes and swollen eyelid; (II) two questions on nasal symptoms—runny nose and stuffed nose; (III) two questions on throat symptoms—dry throat and sore throat; (IV) four questions on skin symptoms—rashes in hands and arms, rashes in face and neck, skin itchiness in hands and arms, and skin itchiness in face and neck; (V) one question on headache; (VI) one question on tiredness. All of these questions had four frequency-related optional answers: “Yes, everyday”, “Yes, 1–4 times every week”, “Yes, 1–3 times every month” or “No, never”. A new binary variable (0/1) was created, representing weekly or more frequent symptoms for each of the six categories of SBS symptoms. An SBS score was calculated by summing up these six binary variables, ranging from 0 to 6.

### 2.3. Methods for Dust Sampling and Measurement of Environmental Characteristics

Dust samples were collected from classrooms in Johor Bahru and Terengganu by vacuuming dust from curtains, floors, bookshelves, chairs, and tables, using a sampler (ALK Abello, Copenhagen, Denmark) with a filter pore size of 6 µm. Dust was vacuumed for four minutes, with two minutes spent on the floor and two minutes on the upper surfaces of desks and chairs (Figure S1). In the school in Penang, the settled dust was collected on the frame of the blackboard with a metal spoon. The vacuumed and settled dust was sieved into fine dust using a metal screen with an aperture of 0.3 mm and stored in a −80 °C freezer. Carbon dioxide (CO_2_), relative air humidity (RH), and room temperature were recorded by Q-track, a direct reading instrument with a data logger (TSI Incorporated, ST Paul, MN, USA). The instrument was calibrated by the local importer in Uppsala prior to the measurements. The instrument was placed at 0.9 m above the floor, in front of the classroom at a minimum 1.5 m distance from the students’ and teachers’ breathing zones. The number of persons in the classroom during the measurement was noted. Data were recorded by the instrument every second, and mean levels over the whole sampling period were calculated. The measurement range of the instrument is 0 to 5000 ppm CO_2_, 5 to 95% RH, and −10 to 60 °C. Accuracy is ±3% of the CO_2_ reading (or ±50 ppm CO_2_) whichever is greater, ±3% RH, and ±0.5 °C temperature. The resolution of the measurement is 1 ppm CO_2_, 0.1% RH, and 0.1 °C temperature. Indoor nitrogen dioxide (NO_2_) was sampled by diffusion sampling for 7 days, with one sampler in each classroom. The samplers were placed on one of the walls in the classroom, about 1.5 m above the floor. The sampler was a badge-type sampler fully based on the theory of diffusion sampling [22]. With such a sampler, the theoretical sampling rate can be used to calculate the pollutant concentrations. The lower detection limit for one week of sampling is 0.4 µg NO_2_/m^3^. The NO_2_ sampler has been compared to active sampling in a routinely managed network [22]. The precision is ±5%. The samplers were prepared and analyzed at an accredited laboratory (IVL Swedish Environment Laboratory).

### 2.4. Microbial Sequencing and Metabolome Profiling

#### 2.4.1. High-Throughput Amplicon Sequencing to Detect Microbial Communities in Dust

Dust samples were used for high-throughput amplicon sequencing for bacterial and fungal identification. Total microbial DNA was extracted from 10 mg of fine dust using the E.Z.N.A soil DNA kit D5625-01 (Omega Bio-Tek, Inc., Norcross, GA, USA) and the Fast DNA SPIN kit (MP Biomedicals, Santa Ana, CA, USA). Fungal ITS (Internal Transcription Spacer) and the bacterial 16S rRNA gene regions were amplified during library preparation and added sample-specific barcode sequences. The amplicons were sequenced using PacBio Sequel and Illumina MiSeq platforms, and the raw sequence data were deposited at QIITA (12875) and Genomic Sequence Archive (CRA002825, CRA002876, CRA005646, and CRA005647). Absolute bacterial and fungal concentrations were quantified via qPCR using universal primers. Two separate extractions of 10 mg dust were analyzed using real-time PCR to determine the absolute concentrations of total bacteria and fungi. The SYBR Green method was employed for universal bacterial detection. The 20 µL reaction mixture comprised 10 µL of Master Mix (Hieff™ qPCR SYBR^®^ Green Master Mix), 2 µL of template DNA, and 0.5 µL of each primer. For bacteria, the primers used were forward: 5′-GCAGGCCTAACACATGCAAGTC-3′ and reverse: 5′-CTGC TGCCTCCCGTAGGAGT-3′ [23]. For fungal detection, the ITS 1 region was targeted, using the forward primer 5.8F1: 5′-AACTTTCAACAACGGATCTCTTGG-3′ and reverse primer 5.8R1: 5′-GCGTTCAAAGACTCGATGATTCAC-3′ [24].

#### 2.4.2. Non-Targeted LC-MS Detection of Metabolites/Compounds in Dust

For profiling chemical compounds in classroom dust, untargeted LC-MS (liquid chromatography–mass spectrometry) was used at BioNovoGene (Suzhou, China). Specifically, 0.6 mL of 2-chlorophenylalanine in methanol was added to 10 mg of fine dust and centrifuged. Furthermore, 300 μL of the supernatant was filtered with a 0.22 μm membrane. LC-MS detection was performed according to the Vanquish HPLC System Q Exactive HF-X Hybrid Quadrupole-Orbital Mass Spectrometer (Thermo Fisher Scientific, Waltham, MA, USA). For electrospray ionization mass spectrometry (ESI-MSn) experiments, positive and negative modes were used for a spray voltage of 3.5 kV and −2.5 kV. Other parameters included 30 arbitrary units of sheath, 10 arbitrary units of auxiliary gas, and a capillary temperature of 325 °C. The parameters of the analyzer were a mass range of *m*/*z* 81–1000 and a mass resolution of 60,000. Chemicals were annotated from the Human Metabolome Database, MassBank, the mzCloud database, METLIN, and MoNA.

### 2.5. Bioinformatics and Statistical Analysis

#### 2.5.1. Screening of Characteristic Microbial Taxa by Region

The QIIME2 platform processed and analyzed microbiome data [25], assigning raw reads to samples based on the barcode information and removing low-quality and chimeric reads. Sequence taxonomy annotation was performed using the Silva (release 138.2) and the UNITE database (release 10.0) [26,27].

A two-step analysis was conducted to identify characteristic microbial taxa associated with SBS symptoms. First, microbial taxa enriched in high SBS and low SBS schools (LDA > 2, *p* < 0.05) were screened using LEfSe analysis [28]. In each center, high SBS and low SBS schools were stratified based on the number of participants with >2 in SBS score. In each center, four schools were defined as high SBS schools, and four schools were defined as low SBS schools. The potential candidate microbial taxa were further assessed by a three-level hierarchical linear regression model adjusted for gender, smoking habit, and parental asthma/allergy (classrooms as the second level and schools as the third level), examining the associations between the abundance of microbial taxa and SBS scores across three centers. The abundance of microbial taxa was normalized by logistic transformation as log10(relative abundance × 105 + 1). FDR correction adjusted *p*-values in the regression analysis to address multiple testing issues and reduce the false discovery rate. This two-step method limited the microbial taxa analyzed in the regression model to 15–35, significantly reducing the risk of false positives.

The association between the alpha diversity of indoor bacteria and fungi was also assessed by three-level hierarchical linear regression models adjusted for gender, smoking habit, and parental asthma/allergy (classrooms as the second level and schools as the third level). Alpha-diversity of indoor bacteria and fungi was calculated by the Shannon diversity index and the observed number of species.

#### 2.5.2. Screening of Potential Protective/Risk Metabolites/Compounds

In each center, significantly enriched compounds (*p* < 0.05, FDR < 0.1, fold change > 2, Mann–Whitney test) were identified in high and low SBS schools. Given that over 100 compounds were initially screened in each center, we conducted further analysis to identify compounds consistently enriched across multiple centers. Specifically, the final identified compounds were enriched in at least two of the three study centers. The associations between the abundance of compounds and SBS scores and specific SBS symptoms were assessed by a three-level hierarchical linear regression model adjusted for gender, smoking habit, and parental asthma/allergy (classrooms as the second level and schools as the third level; *p* < 0.01). The abundance of compounds was calculated from intensity values obtained from untargeted metabolomics, with a log10 transformation applied to normalize the values.

The co-occurrence probabilities of characteristic bacteria and characteristic metabolite interactions were estimated by microbe-metabolite vector analysis (hereinafter referred to as mmvec) [29]. The PubChem database served as a source for key characteristics of chemical compounds.

#### 2.5.3. Mediation Analysis of Other Possible Influencing Factors

Associations between indoor environmental characteristics and SBS score were analyzed using a single-factor linear regression model. Discovering that indoor NO_2_ concentration, CO_2_ concentration, and weight of settled dust were linked to both health outcomes (SBS scores) and health-associated microbial taxa, a causal mediation analysis was conducted to elucidate potential pathways and mediator roles [30]. This analysis was performed with Stata’s medeff command as follows:

medeff (regress M T) (logit Y T M), treat(T) mediate(M) sims(1000) seed(1).(1)

Here, ‘M’ denotes the relative abundance of a microbial taxon or metabolites (mediator), ‘T’ denotes the indoor NO_2_/CO_2_/dust weight (treatment), and ‘Y’ denotes the SBS score.

In this study, all regression models were conducted by StataSE 15.0 (StataCorp LLC, College Station, TX, USA), and other statistics were conducted with IBM SPSS software 21.0 (IBM Corp., Armonk, NY, USA).

## 3. Results

### 3.1. Prevalence of SBS Symptoms Across Three Centers

The study was conducted across three distinct geographical regions in Malaysia: Johor Bahru (South), Terengganu (Northeast), and Penang (Northwest). Within each region, eight junior high schools were randomly selected. Health data were collected through 1139 self-administered questionnaires on SBS symptoms from junior high school students, achieving a participating rate of 89.6%. The prevalence of six specific SBS symptoms, along with the overall SBS scores, was calculated from the sum of the six SBS symptoms (Table 1 and Table S2).

Johor Bahru exhibited the highest prevalence of reported tiredness (23.1%) across the studied regions. Terengganu reported the highest prevalence of eye (24.0%), throat (19.7%), and skin (32.6%) symptoms, while Penang was most affected by nasal symptoms (30.2%) and headaches (26.9%). Despite these differences in symptom prevalence, the overall prevalence rates of SBS symptoms were comparably high across all three centers (Johor Bahru at 51.0%, Terengganu at 54.6%, and Penang at 53.4%). The SBS score in Terengganu was significantly higher than in Johor Bahru (*p* < 0.001, chi-square test) and Penang (*p* = 0.02), suggesting a greater prevalence of multiple SBS symptoms among participants in Terengganu compared to the other two regions.

Furthermore, significant variability in SBS scores was observed within each center (Table S3). For instance, in Johor Bahru, the percentage of participants with an SBS score greater than 2 ranged from 4.65% to 31.82% across different schools. Similarly, this variation was observed in Terengganu (17.86% to 37.66%) and Penang (13.04% to 30.95%), underscoring the significant impact of school-specific environmental characteristics on the occurrence of SBS symptoms.

We further assessed the association between demographic and environmental characteristics and SBS scores. SBS scores did not differ between male and female students across all centers, indicating no gender difference for SBS scores (*p* > 0.05, *t*-test). For environmental characteristics, higher indoor NO_2_ concentration and weight of indoor settled dust were associated with a higher SBS score in Johor Bahru in a linear regression analysis (*p* = 0.001 and 0.006, Table 2). Similarly, a higher indoor CO_2_ concentration was associated with a higher SBS score in Terengganu (*p* = 0.01).

### 3.2. Characteristic Microorganisms Associated with SBS

Alpha-diversity of indoor bacteria and fungi, assessed by the Shannon diversity index and the observed number of species, was not significantly associated with SBS scores across all three centers in Malaysia (*p* > 0.05, linear regression model). The result indicates that microbial alpha-diversity in classrooms is not contributing to the occurrence of SBS symptoms. The relative abundance of the top 10 indoor bacterial and fungal genera in each center is shown in Figure S2.

**Figure 2:**
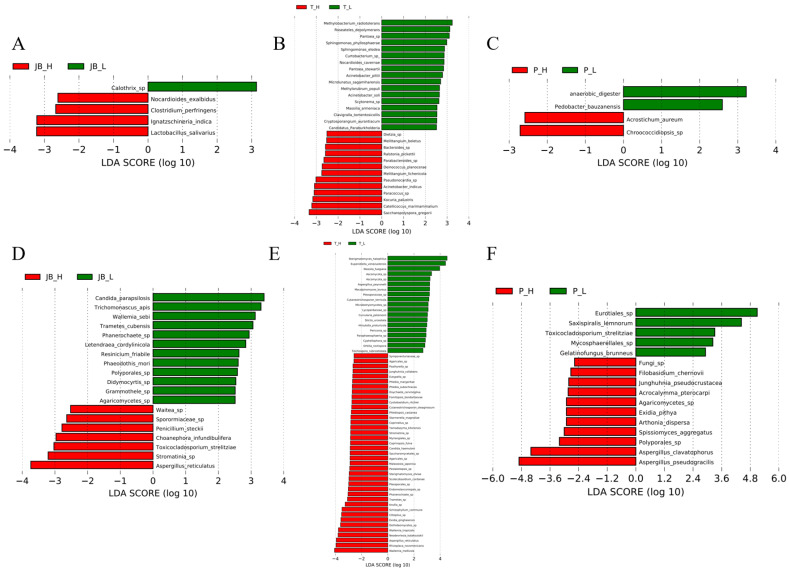
Characteristic microbial profiles of high SBS and low SBS schools in three centers of Malaysia. Characteristic bacterial (**A**) and fungal genera (**D**) in Johor Bahru; characteristic bacterial (**B**) and fungal genera (**E**) in Terengganu; characteristic bacterial (**C**) and fungal genera (**F**) in Penang. Analyses were conducted by LEfSe, and only species microbial taxa with an LDA score > 2.5 and *p* < 0.05 are presented in the figure.

We further identified indoor microorganisms associated with SBS symptoms. Given the high number of indoor microbial taxa (>1000), direct association testing could lead to false positives due to multiple testing issues. Therefore, a two-step screening approach was conducted. First, LEfSe analysis identified indoor microbial taxa enriched in high-SBS and low-SBS schools (Figure 2). Next, the association between the candidate indoor microbiome identified by LEfSe and the SBS score was assessed using a regression model.

Using LEfSe, we identified 1 bacterial and 12 fungal genera/species in low SBS schools, and 4 bacterial and 7 fungal genera/species were identified in high SBS schools in Johor Bahru. In Terengganu, 17 bacterial and 19 fungal genera/species were identified in low SBS schools, and 13 bacterial and 39 fungal genera/species were identified in high SBS schools. In Penang, 2 bacterial and 5 fungal genera/species were identified in low SBS schools, and 2 bacterial and 11 fungal genera/species were identified in high SBS schools. Low SBS school-enriched bacteria mainly from three phyla, including Bacteroidota, Cyanobacteria, and Proteobacteria, whereas high SBS school-enriched bacteria were mainly from four phyla, including Actinobacteriota, Proteobacteria, Deinococcota, and Firmicutes. All identified fungi, whether from low SBS schools or high SBS schools, were from Ascomycota and Basidiomycota.

Most of the identified bacterial and fungal taxa were distinct among the three centers, but some were shared. For example, among the fungal results, *Aspergillus reticulatus* prevailed in high SBS schools of Johor Bahru and Terengganu. There were also contradicted results. For example, *Toxicocladosporium strelitziae* prevailed in Johor Bahru’s high SBS schools and Penang’s low SBS schools.

Subsequently, the association between the candidate indoor microbiome identified by LEfSe and the SBS score was assessed using a three-level linear regression model to identify the most likely SBS-associated indoor microorganisms. Only associations with FDR < 0.05 were reported (Table 3). *Curtobacterium* in Terengganu was negatively associated with SBS (*p* < 0.01, FDR < 0.05). Potential risk bacteria identified in Johor Bahru included *Clostridium perfringens* and *Bacterium 1227R* (*p* < 0.01, FDR < 0.05). In contrast, all identified fungal taxa, including *uc_f_Auriculariaceae*_sp., *Duportella kuehneroides*, and *Wallemia mellicola*, were positively associated with SBS (*p* < 0.01, FDR < 0.05) in both Johor Bahru and Terengganu. Interestingly, these potential risk fungal taxa were all from the phylum of Basidiomycota, suggesting Basidiomycota may contribute to the adverse health effects in SBS.

Overall, distinct SBS-associated bacteria were observed in each center, a finding consistent with previous studies in allergic diseases [31].

### 3.3. Mediation Analysis Between Environmental Characteristics, Indoor Microorganisms, and SBS Symptoms

We further explored how environmental characteristics might affect the abundance of SBS-related indoor microorganisms (Table S1). Specifically, in Johor Bahru, indoor NO_2_ concentration was associated with *uc_f_Auriculariaceae*_sp. (*p* = 0.039). These findings indicate a connection between environmental characteristics, indoor microorganisms, and SBS symptoms. To elucidate the potential pathways and mediator roles, we performed a causal mediation analysis. Our analysis highlighted significant microbial mediation effects in Johor Bahru: the adverse health effects of NO_2_ in Johor Bahru were partially mediated by the increased abundance of *uc_f_Auriculariaceae*_sp. in the city (*p* = 0.028, total effect mediated 51.40%, Table 4).

### 3.4. Characteristic Metabolites Associated with SBS Symptoms

A total of 2633 chemicals were characterized by untargeted LC-MS. Unlike the distinct microbial taxa associated with SBS symptoms across three centers, metabolites showed higher consistent patterns among centers. For example, certain metabolites, including deoxyribonucleosides (S-adenosylmethionine), an indole derivative (N-acetylserotonin), an organonitrogen compound (sphinganine), a quinoline derivative (4-hydroxy-2-quinolone), and (2E,4Z,8E)-Colneleic acid, were enriched in low SBS schools compared to high SBS schools in at least two centers (*p* < 0.01, FDR < 0.05, fold change > 2, Mann–Whitney test; Table 5). These metabolites were classified as potential protective metabolites. To minimize false positives, the association between these metabolites and SBS scores/individual symptoms was further validated in a linear regression model, confirming their protective effects (*p* < 0.01). Specifically, S-adenosylmethionine and N-acetylserotonin had potential protective effects against nasal symptoms, while sphinganine, 4-hydroxy-2-quinolone, and (2E,4Z,8E)-Colneleic acid had potential protective effects against tiredness. These metabolites also have been previously acknowledged for their health benefits, including antioxidant, anti-inflammation, and antibacterial (Table 5).

We further explored the potential microbial sources of these potential protective metabolites. Mmvec analysis showed a co-occurrence probability between these metabolites and the protective indoor microorganisms. For instance, sphinganine was strongly associated with Scytonema and an uncharacterized Armatimonadales in Johor Bahru but associated with Leclercia in Terengganu and associated with Bifidobacteria and Aquamicrobium in Penang (co-occurrence probability value > 1.0).

**Figure 3:**
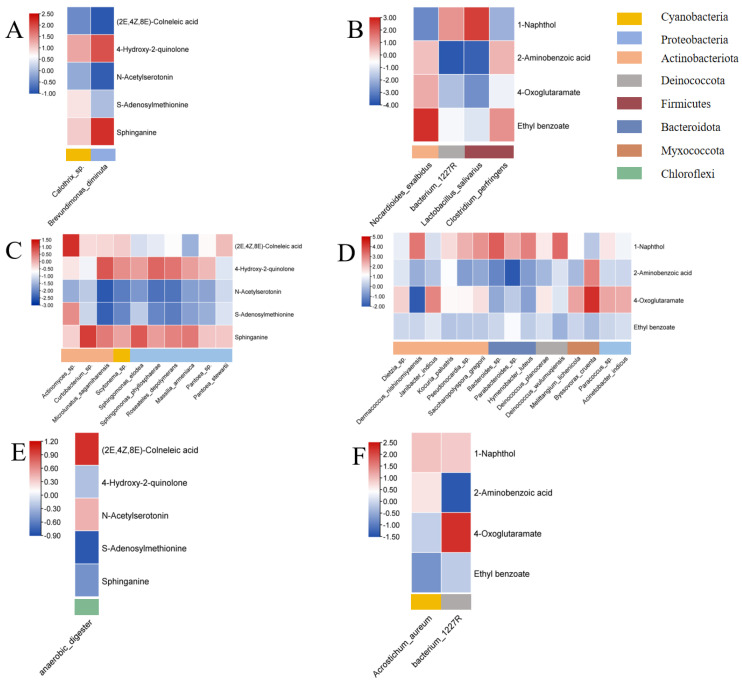
Co-occurrence probabilities between characteristic bacterial taxa and potential characteristic metabolites across three regions of Malaysia. Results are shown for (**A**) low-SBS schools in Johor Bahru (potential protective bacteria and metabolites), (**B**) high-SBS schools in Johor Bahru (potential risk bacteria and metabolites), (**C**) low-SBS schools in Terengganu (potential protective bacteria and metabolites), (**D**) high-SBS schools in Terengganu (potential risk bacteria and metabolites), (**E**) low-SBS schools in Penang (potential protective bacteria and metabolites), and (**F**) high-SBS schools in Penang (potential risk bacteria and metabolites). Bacterial taxa are displayed on the *x*-axis, and metabolites on the *y*-axis. Protective microbial taxa were identified as those enriched in low-SBS schools with an LDA score > 2, while risk microbial taxa were enriched in high-SBS schools with an LDA score > 2. Potential protective or risk metabolites were defined as those significantly enriched (*p* < 0.05, *p* < 0.01, FDR < 0.05, fold change > 2, Mann–Whitney test) in low- or high-SBS schools across at least two regions.

We further explored the bacterial sources of these potential metabolites (Figure 3). Mmvec analysis showed a high co-occurrence probability between these metabolites and bacteria identified in low SBS schools. For instance, sphinganine was strongly associated with *Brevundimonas diminuta* in Johor Bahru and *Curtobacterium* sp. in Terengganu (co-occurrence probability value > 1.0). The (2E,4Z,8E)-Colneleic acid was strongly associated with *Pseudonocardia* sp. in Terengganu and *Anaerobic digester* in Penang.

Potential risk chemicals/metabolites were also identified by Mann–Whitney test and linear regression models (*p* < 0.01, FDR < 0.05, fold change > 2, Mann–Whitney test; *p* < 0.01, linear regression model; Table 5). Among the four potential risk chemicals/metabolites, three were synthetic chemicals, including benzene derivatives (ethyl benzoate, 2-aminobenzoic acid) and 1-naphthol. These compounds were associated with adverse effects in eye and nasal symptoms in this study, and previous studies also reported the adverse effects of these chemicals, including skin, eye, and respiratory tract irritation. We further examined the co-occurrence of high-SBS school bacteria and potential risk metabolites: *Lactobacillus salivarius* was strongly associated with 1-naphthol, and ethyl benzoate was strongly associated with *Nocardioides exalbidus* in Johor Bahru (co-occurrence probability > 1.0). In Terengganu, *Byssovorax cruenta* and uc Bacterium 1227 in Penang were strongly associated with 4-oxoglutaramate (co-occurrence probability > 2.0).

These findings suggest that different centers might have distinct microorganisms producing these metabolites.

## 4. Discussion

The study revealed environmental and microbial factors associated with the prevalence of SBS symptoms across schools in three distinct Malaysian regions. Indoor NO_2_ and CO_2_ concentration and indoor weight of settled dust were identified as potential risk factors for SBS symptoms. Distinct indoor bacterial and Basidiomycota were associated with SBS in three centers. Unlike the distinct microbial association pattern, indoor metabolites/chemicals showed higher consistent patterns among centers: five potential protective and four potential risk metabolites and chemicals were identified in at least two centers in Malaysia. Synthetic chemicals were the main component of potential risk compounds for SBS symptoms. Building upon these findings, there is potential for application in intervention strategies, emphasizing the importance of maintaining a balanced indoor microbiome and mitigating exposure to harmful substances.

### 4.1. Strengths and Limitations of the Study

This study significantly enhances our understanding of SBS by bridging many of the gaps identified in previous investigations. First, unlike prior microbiome-SBS studies, which were confined to single centers or specific geographic locations, this research extends across multiple centers, thereby providing a more comprehensive and generalized perspective on the impact of indoor microbiome on SBS. Second, the study applied cutting-edge high-throughput sequencing techniques. In contrast to the traditional low-throughput methods such as cultivation and quantitative PCR used in the majority of earlier studies [40], this technique has enabled us to obtain a broader and more holistic understanding of the microbial ecosystem. Third, this study employed high-throughput metabolomics approaches, promoting the application of high-throughput metabolomics methods in the field of SBS research. By delving into the role of indoor microbial and synthetic chemicals, this study has shed light on potential contributors to SBS that have remained largely unexplored until now.

Nevertheless, it is important to acknowledge the limitations of this research. First, the dust collection methods used varied between sites, which might introduce a sampling bias. Dust from floors, desks, tables, bookshelves, and curtains was vacuumed in Johor Bahru and Terengganu, and dust was collected from the blackboard frame in Penang. However, as all the sampled areas are seldom or never cleaned, the dust from these different sources should provide a comparable representation of long-term exposure. Second, the application of second-generation amplicon sequencing limited our investigation to the genus level, thus prohibiting species-level differentiation. Another caveat is the exclusive sequencing of the marker gene (16S rRNA and ITS), which impedes the assessment of functional gene abundance and associations. Nonetheless, the shortcomings associated with gene sequencing were mitigated to a certain extent by our comprehensive metabolomic profiling of indoor microbial metabolites, which allowed for a more direct evaluation of microbial functional product exposures. Future research endeavors addressing these limitations could yield more refined insights into SBS and its underlying causes.

### 4.2. Protective and Risk Microorganisms, Metabolites, and Chemicals for SBS

The potential protective and risk indoor bacterial and fungal taxa were identified in this study. However, it is important to note that the majority of these taxa are being reported for the first time in association with SBS. This novel finding limits our ability to discuss their specific roles in the development of SBS with confidence. Epidemiological evidence supporting the protective effects of Cyanobacteria against SBS is sparse, with only one study indicating such associations [3]. The detailed mechanisms and roles of these microorganisms in the pathogenesis or prevention of SBS remain unexplored and warrant further investigation.

In this study, we characterized five metabolites with potential protective effects against SBS, including S-adenosylmethionine, N-acetylserotonin, sphinganine, 4-hydroxy-2-quinolone, and (2E,4Z,8E)-colneleic acid. These metabolites predominantly originated from microorganisms and plants. Most of these metabolites are recognized for their anti-inflammatory, antioxidant, and antibacterial health benefits. For instance, S-adenosylmethionine contributes to immune function, maintains cell membranes, and aids in the breakdown of brain chemicals such as serotonin, melatonin, and dopamine. Studies on S-adenosylmethionine supplementation suggest its potential to mitigate symptoms of depression, osteoarthritis, and liver inflammation and disease [32,33,34]. N-acetylserotonin boasts antioxidant and neuroprotective effects, possibly playing a role in maintaining circadian rhythms [35]. Sphinganine, a type of sphingolipid, is crucial to various cellular functions, including cell signaling and membrane integrity [36]. Quinolones are well-known for their broad-spectrum antibacterial and antioxidant activity [37,38], and colneleic acid, a product of the lipoxygenase pathway, has established roles in plant defense responses [39]. Although most of these metabolites are recognized for their beneficial benefits, none have been previously reported to have a protective association with SBS. However, this study has achieved this. Nevertheless, the protective role of these microbial and plant metabolites in SBS requires further investigation, but these substances could be instrumental in future SBS treatment or intervention strategies.

In addition to these potential protective metabolites, four substances may increase the risk of SBS. These substances are mainly synthetic chemicals (ethyl benzoate, 2-aminobenzoic acid, 1-naphthol). Synthetic chemicals can be irritants or toxic, especially if they become volatile and infiltrate indoor air [31,41,42]. Furthermore, these synthetic chemicals have been linked with adverse health effects in allergic and inflammatory diseases, such as asthma, rhinitis, and several chronic inflammatory diseases [31,43,44], consistent with our findings that they are associated with nasal and eye symptoms.

### 4.3. Mediation Effects of Indoor Microorganisms on Environmental Characteristics and SBS Symptoms

In this study, indoor NO_2_ and CO_2_ concentrations were positively associated with SBS symptoms, which is consistent with previous publications. For example, a study in Taiyuan, China investigated the relationship between indoor and outdoor air pollution and the prevalence, incidence, and remission of SBS symptoms among junior high school students [45]. The study identified NO_2_ levels in classrooms as positively associated with the prevalence of SBS symptoms, indicating that NO_2_ is an important factor in increasing SBS. Research also has found that CO_2_ levels above certain thresholds can lead to symptoms such as dry throat, breathing difficulty, and nose irritation, which are indicative of SBS [14].

In this study, indoor NO_2_ concentration affected the abundance of SBS-related indoor microorganisms. Previous studies reported that environmental pollutants, such as ventilation, temperature, humidity, and the presence of particulate matter, which includes pollutants like NO_2_, can affect microbial concentrations in indoor environments [46,47]. Specifically, two studies in Malaysia also reported that indoor NO_2_ concentrations were associated with the overall bacterial community variation of infection-related indoor microorganisms [24,48]. These studies indicate that indoor NO_2_ concentration affects the composition and diversity of microbial communities in built environments.

Given the interconnected associations between environmental characteristics, indoor microorganisms, and SBS, we conducted causal mediation analysis. Our investigation revealed significant microbial mediation effects: notably, the detrimental health impacts of NO_2_ exposure and the total weight of dust on SBS in Johor Bahru were found to be partially mediated through the elevated presence of the *uc_f_Auriculariaceae*_sp. This finding marks a documented instance of an indoor microorganism mediating the effects of environmental pollutants on SBS. By identifying specific microorganisms that mediate health outcomes, this research opens new avenues for mitigating SBS through targeted interventions.

### 4.4. Microorganisms and Metabolites for SBS Intervention Strategies

Our study has provided novel insights into the potential role of microorganisms and their metabolites in SBS. Significant microbial variations were observed across different centers in Malaysia [30], each boasting unique protective and risk microorganisms for SBS. Furthermore, our findings were corroborated by another study in urban and rural schools of Shanxi province, which showed the protective and risk microorganisms for SBS in Shanxi differed from those in Malaysia [2]. These observations indicate that protective and risk microorganisms for SBS vary geographically, thus highlighting the complex interplay of geographical, environmental, and microbial factors in the manifestation of SBS. However, the consistent protective role of certain microbial metabolites across all centers in Malaysia underscores their potential as future intervention strategies. Interestingly, the majority of these protective metabolites could originate from microorganisms themselves, suggesting that indoor microbial communities may be a source of bioactive compounds with potential health benefits. This is reminiscent of the human gut microbiome, where microbial metabolites have been shown to significantly impact human health and disease [49,50].

We also identified potential risk substances predominantly consisting of synthetic chemicals. This suggests that minimizing exposure to these substances could be an effective strategy for SBS prevention. However, further research is needed to understand the specific exposure thresholds associated with SBS risk and to validate these findings in different indoor environments.

Overall, our results suggest a dual strategy for SBS intervention: On the one hand, cultivating a healthy indoor microbiome to enhance the production of protective metabolites; on the other hand, reducing exposure to identified risk substances. While individual microorganisms may be replaceable, maintaining a diverse microbial community capable of producing the identified protective metabolites seems to be an important strategy. The development of effective interventions will likely require a multifaceted approach, addressing both the indoor microbiome and the chemical landscape, as well as interactions with host factors [11,51]. The findings from this study provide a solid foundation for further investigations into these potentially promising intervention strategies for SBS.

## 5. Conclusions

In this multicenter study, we investigated the relationship between indoor microbial taxa, metabolites, and SBS symptoms in Malaysian schools. Our findings highlight the significant prevalence of SBS symptoms among students and the variation in prevalence among schools. Distinct indoor bacterial and fungal taxa and metabolites were identified as either protective or risk factors for SBS symptoms, varying across different regions. Metabolites, predominantly derived from environmental microorganisms, showed potential health benefits such as anti-inflammatory, antioxidant, and antibacterial properties. Conversely, certain synthetic chemicals were linked to higher SBS symptoms, emphasizing the need to minimize exposure to these harmful substances. Mediation analysis revealed that the adverse health effects of NO_2_ and dust weight on SBS were partially mediated by the increased abundance of specific microorganisms such as *uc_f_Auriculariaceae*_sp., underscoring the complex interplay between environmental pollutants, indoor microbial communities, and health outcomes. This study underscores the importance of maintaining a balanced indoor microbiome and reducing exposure to deleterious substances to mitigate SBS symptoms. The findings provide valuable insights for developing targeted intervention strategies to improve indoor public health, particularly in school environments. Future research should continue to explore the functional roles of indoor microbial communities and their metabolites in relation to SBS, aiming to refine and validate these intervention strategies across diverse geographic locations and settings.

## Figures and Tables

**Table 1:** Prevalence of SBS symptoms in three centers of Malaysia.

Center	Johor Bahru (N = 308)		Terengganu (N = 463)		Penang (N = 368)		Total (N = 1139)	
Symptoms	Number	Prevalence (%)	Number	Prevalence (%)	Number	Prevalence (%)	Number	Prevalence (%)
Eye symptoms	39	12.7	111	24.0	71	19.3	221	19.4
Nasal symptoms	61	19.8	132	28.5	111	30.2	304	26.7
Throat symptoms	52	16.9	91	19.7	64	17.4	207	18.2
Skin symptoms	37	12.0	151	32.6	17	4.6	205	18.0
Headache	60	19.5	110	23.8	99	26.9	269	23.6
Tiredness	71	23.1	101	21.8	83	22.6	255	22.4
SBS score								
0	151	49.0	210	45.4	170	46.2	531	46.6
1	72	23.4	60	13.0	69	18.8	201	17.6
2	36	11.7	64	13.8	53	14.4	153	13.4
3	29	9.4	59	12.7	45	12.2	133	11.7
≥4	20	6.5	70	15.1	31	8.4	121	10.6
Prevalence of SBS score > 2 in schools Mean (Range) (%) ^a^	15.91 (4.65~31.82)		27.86 (17.86~37.66)		20.65 (13.03~30.95)		22.30 (4.65~37.66)	

^a^: This table presents the composition of SBS symptoms within each center. The bottom part shows the mean prevalence of SBS score > 2 and its range by center compared with each other.

**Table 2:** Associations between indoor environmental characteristics and SBS score in three centers of Malaysia.

Center	Environmental Characteristics	Median (Q1–Q3)	Β (95% CI)	*p* Value ^a^
Johor Bahru	Indoor CO_2_ concentration (ppm)	512.0 (410.0~537.0)	0.00 (0.00~0.00)	0.27
	Indoor relative humidity (%)	71.5 (65.3~72.7)	0.01 (−0.02~0.05)	0.44
	Indoor NO_2_ concentration (μg/m^3^)	19.5 (16.9~29.3)	0.03 (0.01~0.05)	**0.001**
	Age of building (years)	7.0 (4.0~18.0)	0.00 (−0.01~0.01)	0.81
	Weight of settled dust (g)	1.2 (0.9~1.6)	0.35 (0.10~0.61)	**0.006**
Terengganu	Indoor CO_2_ concentration (ppm)	426.0 (413.5~447.0)	0.01 (0.00~0.01)	**0.010**
	Indoor relative humidity (%)	71.6 (70.3~73.6)	0.04 (−0.01~0.09)	0.08
	Indoor NO_2_ concentration (μg/m^3^)	8.9 (7.9~9.7)	−0.07 (−0.10~0.00)	**0.040**
	Weight of settled dust (g)	1.1 (0.89~1.26)	−0.33 (−0.87~0.21)	0.23
Penang	Indoor CO_2_ concentration (ppm)	391.5 (380~415.3)	0.00 (0.00~0.00)	0.43
	Indoor relative humidity (%)	76.4 (74.3~83.9)	0.00 (0.02~0.03)	0.84
	Indoor NO_2_ concentration (μg/m^3^)	22.9 (19.6~27.0)	0.01 (−0.02~0.04)	0.57
	Weight of settled dust (g)	3.21 (2.4~4.5)	0.01 (−0.06~0.08)	0.78

^a^: The environmental characteristics of each center were analyzed by regression analysis with the SBS score of the subjects in each center. Associations with a *p*-value < 0.01 were deemed statistically significant and highlighted in bold. Significant *p*-values were reported to three decimal places, while non-significant *p*-values were rounded to two decimal places.

**Table 3:** Associations between indoor microorganisms and SBS score in three centers of Malaysia.

Center	Kingdom	Phylum	Species	Average Relative Abundance (%)	Β (95% CI)	*p* Value ^a^	FDR ^a^
Johor Bahru	Bacteria	Deinococcota	*Bacterium_1227R*	0.0011	2.58 (1.55~3.61)	<0.001	0.005
		Firmicutes	*Clostridium_perfringens*	0.0008	1.11 (0.40~1.81)	0.002	0.033
	Fungi	Basidiomycota	*uc_f_Auriculariaceae*_sp.	0.0003	1.04 (0.46~1.63)	0.001	0.022
		Basidiomycota	*Duportella_kuehneroides*	0.0004	1.81 (0.63~3.00)	0.003	0.040
Terengganu	Bacteria	Actinobacteriota	*Curtobacterium*_sp.	0.0016	−1.45 (−2.18~−0.73)	<0.001	0.009
	Fungi	Basidiomycota	*Wallemia_mellicola*	0.0150	0.47 (0.19~0.75)	0.001	0.044

^a^: Associations were evaluated using three-level hierarchical linear regression models, adjusted for gender, smoking habits, and parental asthma/allergy history, with classrooms as the second level and schools as the third level. To account for multiple testing and reduce the false discovery rate (FDR), *p*-values were adjusted using FDR correction. Only associations with an FDR < 0.05 were considered significant and included in the table. Non-significant results were omitted.

**Table 4:** Mediating effects of characteristic microorganisms on environmental characteristics and SBS score.

Center	Treatment	Coefficient		Mediator	Coefficient		Total Effect Mediated (%) ^a^
		Β (95% CI)	*p* Value		Β (95% CI)	*p* Value	
Johor Bahru	Indoor NO_2_ concentration	0.01 (−0.01~0.04)	0.24	*uc_f_Auriculariaceae*_sp.	0.80 (0.09~1.51)	**0.028**	51.40%
Johor Bahru	Weight of settled dust	0.34 (−0.10~0.78)	0.13	*uc_f_Auriculariaceae*_sp.	0.65 (−0.12~1.42)	0.10	41.55%

^a^: This table shows the mediating role of characteristic microorganisms in the relationship between environmental characteristics (including indoor NO_2_ concentrations and weight of settled dust) and SBS score. The level of significance is *p* < 0.05. Significant *p*-values were reported to three decimal places, while non-significant *p*-values were rounded to two decimal places.

**Table 5:** Potential protective and risk environmental metabolites identified in three centers of Malaysia.

Potential Protective Metabolites ^a^	Enriched Centers	Associated Symptoms	*p* Value	FDR	Class	Sub Class	Reported Health Effects ^b^
S-Adenosylmethionine	JB and T	Nose (T)	0.010	0.040	5′-deoxyribonucleosides	5′-deoxy-5′-thionucleosides	maintains immune function and cell membranes, anti-inflammation [32,33,34]
N-Acetylserotonin	JB and T	Nose (T)	<0.001	0.001	Indoles and derivatives	Hydroxyindoles	antioxidant, neuroprotective, circadian rhythm [35]
Sphinganine	JB and P	Tiredness (JB)	0.008	0.040	Organonitrogen compounds	Amines	necessary for cellular function under normal physiological conditions [36]
4-Hydroxy-2-quinolone	JB and P	Tiredness (JB)	0.005	0.020	Quinolines and derivatives	Quinolones and derivatives	antibacterial [37,38]
(2E,4Z,8E)-Colneleic acid	JB and P	Tiredness (JB)	0.001	0.010	Fatty Acyls	Fatty acids and conjugates	plant defense responses [39]
**Potential Risk Metabolites**	**Enriched centers**	**Associated symptoms**	***p* value**	**FDR**	**Class**	**Sub Class**	**Reported health effects**
Ethyl benzoate	T and P	Eye (P)	0.001	0.010	Benzene and substituted derivatives	Benzoic acids and derivatives	irritation
2-Aminobenzoic acid	T and P	Nose (P)	0.005	0.020	Benzene and substituted derivatives	Benzoic acids and derivatives	skin and eye irritation
1-Naphthol	T and P	Eye (T)	0.005	0.020	Naphthalenes	Naphthols and derivatives	eye, skin, and respiratory tract irritation
4-Oxoglutaramate	T and P	Nose (T)	0.008	0.040	Keto acids and derivatives	Short-chain keto acids and derivatives	unknown

^a^. Metabolites were defined as protective if they were enriched in low SBS schools compared with high SBS schools (*p* < 0.05, *p* < 0.01, FDR < 0.05, fold change > 2, Mann–Whitney test) in at least two centers. Additionally, these metabolites were negatively associated with at least one symptom based on linear logistic regression analysis (*p* < 0.01). ^b^. Reported health effects of the metabolites/chemicals were retrieved by querying the PubMed and PubChem databases.

## Data Availability

Raw sequence data is available at the QIITA microbial study management platform (https://qiita.ucsd.edu; 12875) and the Genome Sequence Archive (https://ngdc.cncb.ac.cn/gsa; CRA002825, CRA002876, CRA005646 and CRA005647).

## Supplementary Materials

The following supporting information can be downloaded at: https://www.mdpi.com/article/10.3390/metabo15020111/s1, Figure S1: Photographs of projects in schools in Malaysia; Figure S2: Composition of bacterial and fungal taxa at the class level in each center; Figure S3: Characteristic microbial profiles of high SBS and low SBS schools in three centers of Malaysia; Table S1: Regression between environmental characteristics and characteristic microorganisms; Table S2: Demographic data of the three centers and the results of chi-square test; Table S3: The high SBS schools and low SBS schools on the basis of grouping and results; Table S4: Logistic regression was performed between the metabolites with between-group differences and the prevalence of each SBS symptom in each city; Questionnaire.

## Abbreviations

SBS	sick building syndrome
FDR	false discovery rate
GABA	gamma-aminobutyric acid
SCFAs	short-chain fatty acids
LPS	lipopolysaccharide
MVOCs	microbial volatile organic compounds
LEfSe	linear discriminant analysis effect size
Mmvec	microbe-metabolite vectors analysis
LDA	linear discriminant analysis
