# Supplementary material for: A Multicenter Exploration of Sick Building Syndrome Symptoms in Malaysian Schools: Indoor Pollutants, Microbial Taxa, and Metabolites
Source: Metabolites. 2025 Feb 10;15(2):111. doi: 10.3390/metabo15020111 (PMC11857348; doi:10.3390/metabo15020111)
Supplement: Supplementary file 1 [file metabolites-15-00111-s001.zip › metabolites-3452540-supplementary.pdf]

Table S1:Regression between environmental characteristics and characteristic microorganisms. Only the results of the P<0.05 are shown in the following table.

Center	Environmental characteristics	Species	β（95%CI）	P value
Johor Bahru	Indoor NO2 concentration	*bacterium_1227R*	0.0075(-0.0014~0.016)	0.093
		Clostridium_perfringens	0.014(-0.0029~0.031)	0.096
		uc_Auriculariaceae_sp	0.018(0.0011~0.035)	0.039
		Duportella_kuehneroides	0.00073(-0.012~0.014)	0.902
	Weight of settled dust	*bacterium_1227R*	0.010(-0.051~0.25)	0.179
		Clostridium_perfringens	0.036(-0.28~0.36)	0.808
		uc_Auriculariaceae_sp	0.38(0.11~0.65)	0.01
		Duportella_kuehneroides	0.0096(-0.19~0.21)	0.917
Tereng ganu	Indoor CO2 concentration	*Curtobacterium_sp.*	-0.0010(-0.0032~0.0011)	0.34
		Wallemia_mellicola	0.00072(-0.0060~0.0074)	0.826

Note: Regression analysis results between environmental features associated with SBS and characteristic microbes associated with SBS were used to explore the possibility of mediating effects of environmental features on characteristic microbes.

Table S2: Demographic data of the three centers and the results of chi-square test.

Demographic characteristics	Results of classification	Total (N=1139)		JB (n=308)		P (n=368)		T (n=463)		p-value
		number	Percent	number	Percent	number	Percent	number	Percent	
gender	Male	492	43.2	144	46.75	168	45.65	180	38.88	0.049
	Female	647	56.8	164	53.25	200	54.35	283	61.12	
Race(Total number=676)	Chinese	184	27.22	130	42.21	54	14.67			<0.01
	Indian	112	16.57	56	18.18	56	15.22			
	Malay	380	56.21	122	39.61	258	70.11			
Parents have asthma/allergies	no	854	74.98	241	78.25	278	75.54	335	72.35	0.172
	yes	285	25.02	67	21.75	90	24.46	128	27.65	
Someone is smoking at home	no	535	46.97	184	59.74	189	51.36	162	34.99	<0.01
	yes	604	53.03	124	40.26	179	48.64	301	65.01	

Note: Because Terengganu's population was all from Malaysia, ethnic information about the city's population was not included in the group comparison.

Table S3: The high SBS schools and low SBS schools on the basis of grouping and results.

city	Group	Class	SBSscore>2 students	prevalence
Johor Bahru	high	JB-4	10	16.95%
		JB-7	8	18.18%
		JB-8	14	31.82%
	low	JB-1	2	4.65%
		JB-3	6	10.71%
		JB-5	7	14.89%
		JB-6	2	13.33%
Terengganu	high	T-1	20	36.36%
		T-3	13	28.89%
		T-5	13	28.26%
		T-8	29	37.66%
	low	T-2	15	23.81%
		T-4	10	17.86%
		T-6	17	25.37%
		T-7	12	22.22%
Penang	high	P-1	14	25.00%
		P-3	7	20.00%
		P-7	13	30.95%
		P-8	14	20.29%
	low	P-2	9	17.31%
		P-4	3	13.04%
		P-5	9	19.15%
		P-6	7	15.91%

Table S4. Logistic regression was performed between the metabolites with between-group differences and the prevalence of each SBS symptom in each city, grouped by prevalence of each SBS symptom.

Enriched areas	metabolites	symptoms	class	P.value_T	P.value_JB	P.value_P
rural	(2E,4Z,8E)-Colneleic acid	tired	Fatty Acyls		0.001306022	0.039563099
	16(R)-HETE	headache	Fatty Acyls		0.024627083	0.000759885
	1D-Myo-inositol 1,4,5,6-tetrakisphosphate	sbs2	Organooxygen compounds	0.018807102		0.000489889
	2-O-(alpha-D-Mannosyl)-D-glycerate	tired	Carboxylic acids and derivatives		0.008076039	0.01849494
	3-(3,4-Dihydroxy-5-methoxy)-2-propenoic acid	tired	Cinnamic acids and derivatives	0.018215361	0.00310164	
	3-(3,4-Dihydroxy-5-methoxy)-2-propenoic acid	eye	Cinnamic acids and derivatives	0.018215361	0.005959526	
	3,4-Dihydroxyphenylglycol	nose	Phenols	0.007544786		0.004223289
	3-tert-Butyl-5-methylcatechol	nose	NULL	0.004289458		0.012050601
	4-Hydroxy-2-quinolone	tired	NULL		0.005060181	0.016700026
	4-Hydroxyazobenzene	headache	Azobenzenes		0.006357565	0.027859474
	Allopregnanolone	tired	Steroids and steroid derivatives		0.022089056	0.003754146
	All-trans-13,14-dihydroretinol	tired	Fatty Acyls	0.007898867	0.016770456	
	Aminoadipic acid	eye	Carboxylic acids and derivatives		0.013427602	0.007207009
	Benzene-1,2,4-triol	eye	Benzene and substituted derivatives	0.026853873		0.008603155
	beta-D-Glucosamine	tired	Carbohydrates and carbohydrate conjugates		0.000754533	0.027468012
	D-Fructose	eye	NULL	0.002289194	0.042582933	
	Fenofibrate	tired	Benzene and substituted derivatives		0.00310164	0.000563634
	gamma-Glutamylcysteine	skin	Carboxylic acids and derivatives		0.019269934	0.009525262
	Gluconolactone	tired	Organooxygen compounds	0.043709314	0.000426273	
	GMP	skin	Purine nucleotides		0.00939216	0.008059108
	Guanosine	eye	Purine nucleosides		0.002463592	0.013445947
	Guanosine	tired	Purine nucleosides		0.000625312	0.030226255
	Jasmonic acid	skin	Fatty Acyls	0.007898867		0.001900053
	L-Histidine	tired	Carboxylic acids and derivatives		0.02881823	0.000368598
	L-Histidine trimethylbetaine	eye	Carboxylic acids and derivatives		0.001401002	0.000126021
	L-Serine	skin	Carboxylic acids and derivatives		0.000426273	0.004794525
	L-Threonine	tired	Carboxylic acids and derivatives		0.016770456	0.007043985
	Lycorine	nose	NULL	0.003706505	0.032798814	
	Mesaconate	sbs2	NULL	0.005711143	0.001859604	
	Morpholine	eye	Oxazinanes		0.001401002	0.012050601
	Morpholine	tired	Oxazinanes		0.000350637	0.003490317
	myo-Inositol	eye	Organooxygen compounds		0.007050608	0.020556393
	N(omega)-Hydroxyarginine	skin	Carboxylic acids and derivatives		0.037242545	0.008059108
	N-Acetylneuraminate	tired	Carboxylic acids and derivatives		0.000350637	0.039971236
	N-Acetylserotonin	nose	Indoles and derivatives	0.000109064	0.021868088	
	Ophthalmate	tired	NULL		0.000287702	0.01356336
	Prostaglandin F1a	tired	Fatty Acyls		0.00939216	0.007043985
	S-Adenosylmethionine	nose	NULL	0.009889784	0.012298217	
	Sphinganine	tired	Organonitrogen compounds		0.008076039	0.012199755
	Uric acid	tired	Imidazopyrimidines		0.02881823	0.006286587
urban	(13E)-11a-Hydroxy-9,15-	throat	Fatty Acyls		0.021868088	0.000176801

dioxoprost-13-enoic acid					
(2R,3R)-3-Methylornithinyl-N6-lysine	sbs2	NULL		0.002621822	0.000319085
(S)-Abscisic acid	eye	Prenol lipids		0.009785073	0.014982193
1,2-Epoxy-p-menth-8-ene	throat	Oxepanes		0.042445238	0.000130547
1,3,7-Trimethyluric acid	nose	Imidazopyrimidines	0.030382236		0.0032945
1D-chiro-Inositol	eye	NULL		0.032444668	0.005383661
1-Naphthol	eye	Naphthalenes	0.005048259		0.030746779
2,3,4,6-Tetrahydroxybenzophenone	eye	Benzene and substituted derivatives		0.000141486	0.005383661
2-Aminobenzoic acid	nose	Benzene and substituted derivatives	0.015375343		0.00483019
2-Hydroxycinnamic acid	sbs2	Cinnamic acids and derivatives		0.04218801	0.004987742
4-Oxoglutaramate	nose	NULL	0.007544786		0.045002105
4-Phenylbutyrate	sbs2	Benzene and substituted derivatives		0.019269934	0.003490317
5,7-Dihydroxyflavone	throat	Flavonoids		0.009083934	0.036462232
8-Amino-3,8-dideoxy-D-manno-octulosonate	sbs2	NULL		0.005060181	0.000238157
9,10-12,13-Diepoxyoctadecanoate	nose	Carboxylic acids and derivatives	0.034089975		0.001727595
alpha-Tocopherol	nose	Prenol lipids	0.016602338		0.001153105
Anhydroglycinol	throat	NULL		0.032798814	0.002128814
Cholesterol	tired	Steroids and steroid derivatives	0.002693548	0.032799447	
Creatine	throat	Carboxylic acids and derivatives	0.004329091	0.037357745	
Dantron	eye	Anthracenes		0.007050608	0.020556393
D-Glucuronic Acid	nose	NULL	0.00202611		0.001727595
Estradiol	throat	Steroids and steroid derivatives		0.028724859	0.003936546
Estragole	throat	Phenol ethers		0.048106828	0.003490317
Ethyl benzoate	eye	Benzene and substituted derivatives	0.038822846		0.001004876
Ethylmethylacetic acid	throat	Fatty Acyls		0.002047732	0.001448973
Ethylmethylacetic acid	sbs2	Fatty Acyls		4.33164E-05	0.001448973
Formononetin	nose	Isoflavonoids	0.000206304		0.001372286
Galactose 1-phosphate	eye	Organooxygen compounds		0.005959526	0.007667779
Galactose 1-phosphate	tired	Organooxygen compounds		0.002621822	0.01849494
Galipine	sbs2	Azacyclic compounds	0.027026385		0.007882337
Gemcitabine	sbs2	Pyrimidine nucleosides	0.021263814		0.006286587
Geranic acid	eye	Prenol lipids		0.009785073	0.040997061
Guanosine	nose	Purine nucleosides	0.007586665		0.001372286
L-Aspartic acid	sbs2	Carboxylic acids and derivatives	0.009889784		0.024929681
Levorphanol	throat	Morphinans		0.002903145	0.01356336
L-Octanoylcarnitine	sbs2	Fatty Acyls		0.002621822	1.59328E-05
Myristoleic acid	nose	Fatty Acyls	0.001733579		0.003732715
N-a-Acetylcitrulline	headache	Carboxylic acids and derivatives		0.036916336	0.002555566
N-Amidino-L-glutamate	nose	NULL	0.00202611		0.002555566
Perillyl alcohol	nose	Prenol lipids		0.037357745	0.002555566
Phenylbutazone	sbs2	Benzene and substituted derivatives		0.032799447	0.000647614
Quinine	throat	Cinchona alkaloids		0.021868088	0.001271204
Tartaric acid	eye	Organooxygen compounds		0.001695897	0.000494381
Tartaric acid	tired	Organooxygen compounds		0.002621822	0.007043985
Tripelennamine	throat	Benzene and substituted derivatives		0.042445238	0.004433991

	Ubiquinone-1	sbs2	NULL	0.012610956	0.000152026

Figure S1: Photographs of projects in schools in Malaysia.

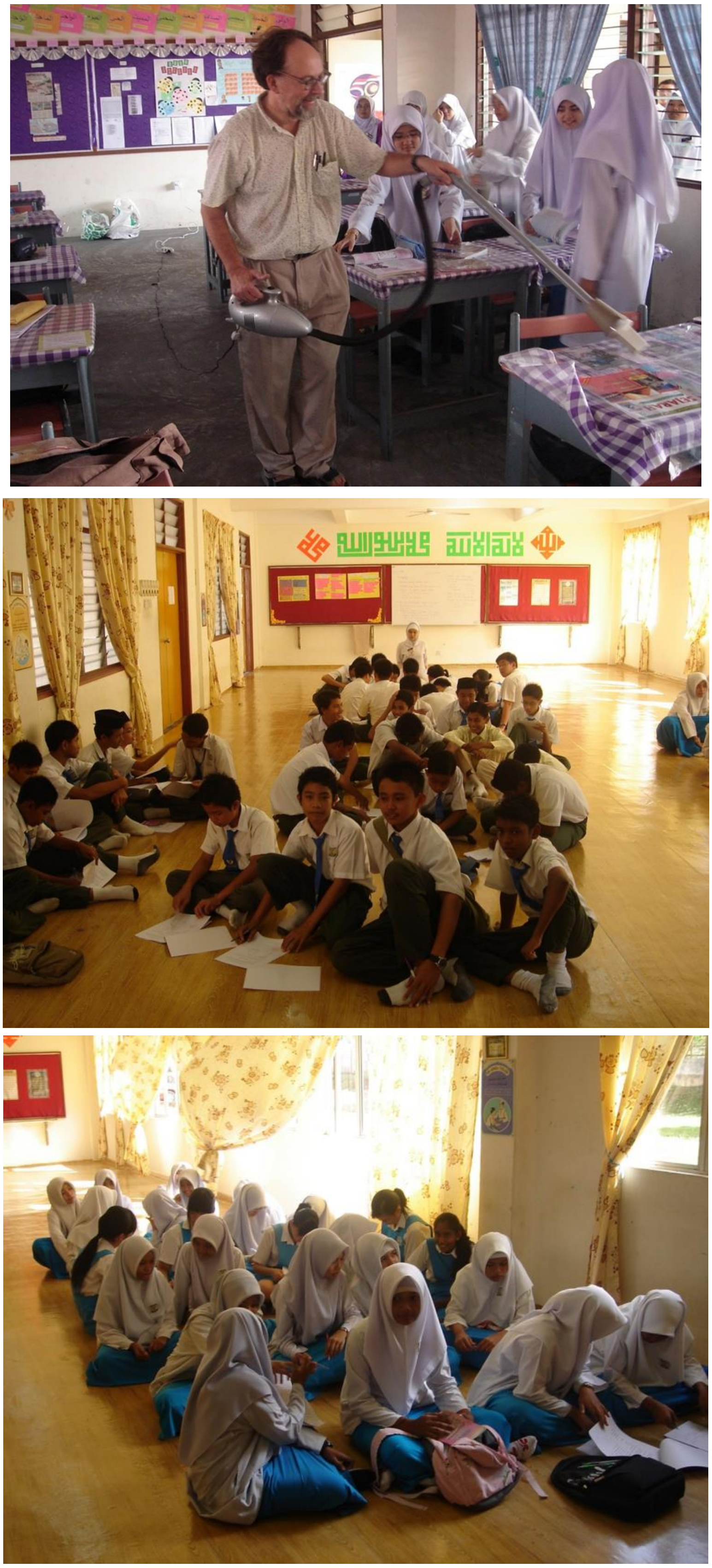

Figure S2: Composition of bacterial and fungal taxa at the class level in each center. (A) Bacterial class composition in Johor Bahru (B) Fungal class composition in Johor Bahru (C) Bacterial class composition in Terengganu (D) Fungal class composition in Terengganu (E) Bacterial class composition in Penang (F) Fungal class composition in Penang.

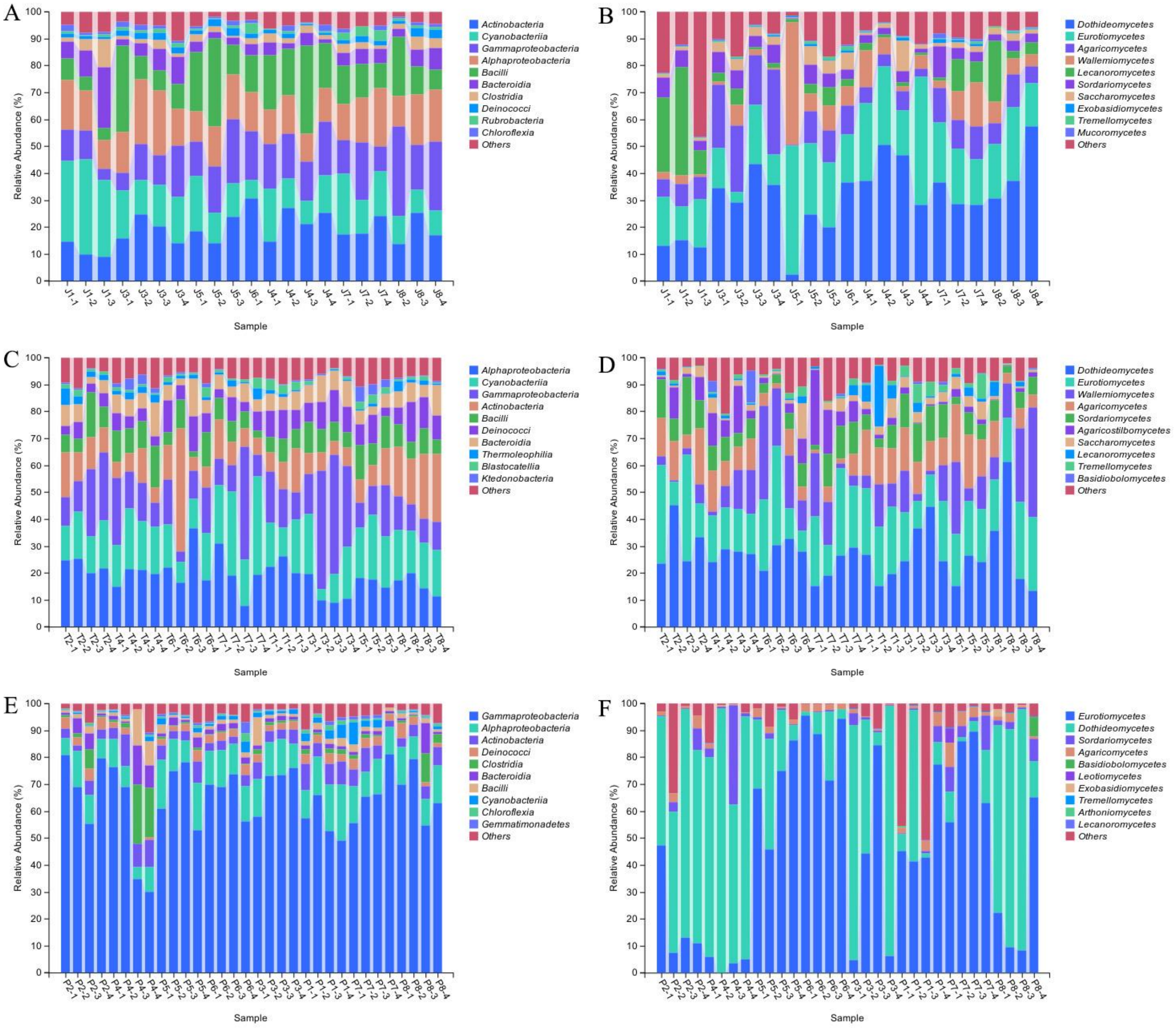

Figure S3: Characteristic microbial profiles of high SBS and low SBS schools in three centers of Malaysia. Characteristic bacterial (A) and fungal genera (B) in Johor Bahru; characteristic bacterial (C) and fungal genera (D) in Terengganu; characteristic bacterial (E) and fungal genera (F) in Penang. Analyses were conducted by LEfSe, and only genus microbial taxa with LDA score > 3 and $p < 0.05$ is presented in the figure.

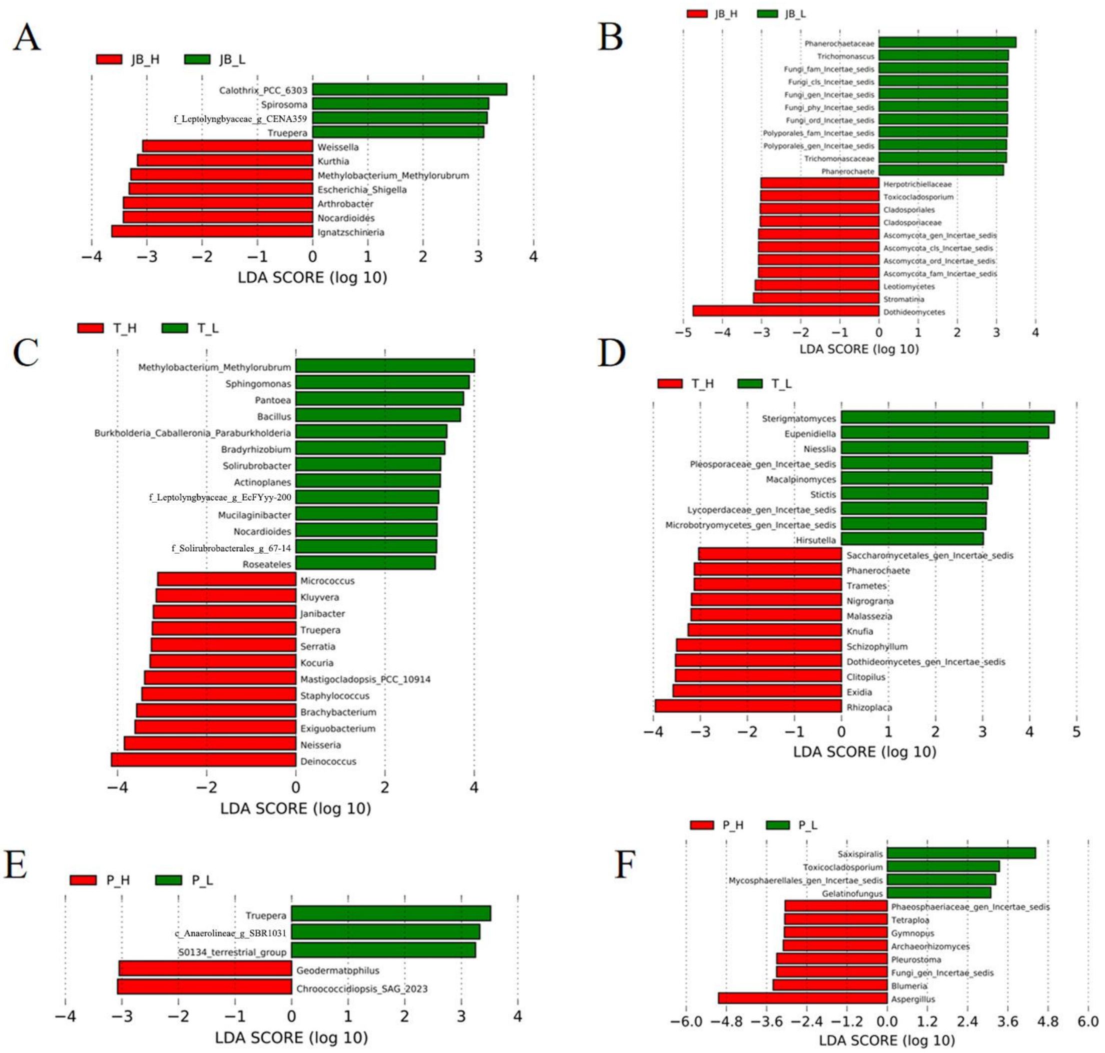

Questionnaire
Junior high school pupils i Shanghai

THE SCHOOL AND HOME ENVIRONMENT, AND YOUR HEALTH

Name________________________________ Age __________________ years

School_______________________________ Classroom_________________

Gender _____________

WHEEZING OR WHISTLING BREATH

1. Have you ever had wheezing or whistling in the chest at any time in the past?
❑ *Yes*
❑ *No*

If you have answered "NO", please skip to question 6.

2. Have you had wheezing or whistling sounds in the chest in the last 12 months?
❑ *Yes*
❑ *No*

If you have answered "NO" please skip to question 6.

3. How many attacks of wheezing have you had in the last 12 months?
❑ *None*
❑ *1 - 3 episodes*
❑ *4 - 12 episodes*
❑ *More than 12 episodes*

4. In the last 12 months, how often, on average, have your sleep been disturbed due to wheezing?
❑ *never woken with wheezing*
❑ *less than one night per week*
❑ *one or more nights per week*

5. In the last 12 months has wheezing ever been severe enought to limit your speech to only one or two words at a time between breaths?

❑ *Yes*
❑ *No*

6. Have you ever had asthma?

❑ *Yes*
❑ *No*

7. In the last 12 months, has your chest sounded wheezy after exercise?

❑ *Yes*
❑ *No*

8. In the last 12 months have you had a dry cough at night, apart from a cough associated with a cold or a chest infection?

❑ *Yes*
❑ *No*

DERMAL SYMPTOMS

1. Have you ever had an itchy rash which was coming and going for at least 6 months?

❑ *Yes*
❑ *No*

If you have answered "NO" please skip to question 7.

---

2. Have you had this itchy rash at any time in the last 12 months?

❑ *Yes*
❑ *No*

If you have answered "NO" go directly to question 7.

---

3. Has this itchy rash at any time affected any of the following places the folds of the elbow, behind the knees, in front of the ankles, under the buttocks, or around the neck, ears or eyes:

- ❑ *Yes*
- ❑ *No*

4. At what age did this itchy rash first occur?

- ❑ *Under 2 years*
- ❑ *Age 2-4*
- ❑ *Age 5 or more*

5. Has this rash cleared completely at any time during the last 12 months?

- ❑ *Yes*
- ❑ *No*

6. <u>In the last 12 months</u>, how often, on average, have you been kept awake at night by this itchy rash?

- ❑ *Never in the last 12 months*
- ❑ *Less than one night per week*
- ❑ *One or more night per week*

7. Have you <u>ever</u> had eczema?

- ❑ *Yes*
- ❑ *No*

NASAL SYMPTOMS

1. Have you <u>ever</u> had a problem with sneezing, or a runny nose, blocked nose when you DID NOT have a cold or the flue?

- ❑ *Yes*
- ❑ *No*

If you have answered "NO" please skip to question 6.

----

2. In the past 12 months have you had a problem with sneezing, or a runny or a blocked nose when you DID NOT have a cold or the flue?

- ❑ *Yes*
- ❑ *No*

3. In the past 12 months, has this nose problem been accompanied by itchy-watery eyes?

- ❑ *Yes*
- ❑ *No*

4. In which of the past 12 months did this nose problem occur (please tick any which apply)

January	❑	*May*	❑	*September*	❑
February	❑	*Jun*	❑	*October*	❑
March	❑	*July*	❑	*November*	❑
April	❑	*August*	❑	*December*	❑

5. In the past 12 months, how much did this nose problem interfere with your daily activities?

- ❑ *Not at all*
- ❑ *A little*
- ❑ *A Moderate account*
- ❑ *A lot*

---

6. Have you ever had hay fever?

- ❑ *Ja*
- ❑ *Nej*

MORE QUESTIONS ABOUT ASTHMA AND ALLERGIES FROM THE UPPSALA QUESTIONNAIRE

Wheezing and whistling in the chest

1. Have you had wheezing or whistling in the chest at any time in **the last 12 months**?________________________ Yes ( ) No ( )

If "no" go to question 5, if "yes":

2.Have you been at all breathless when the wheezing noise was present? ________ Yes ( ) No ( )

3.Have you had this wheezing or whistling when you did *not* have a cold? ________ Yes ( ) No ( )

4.Have you woken up with a feeling of tightness in the chest in **the last 12 months**? ________ Yes ( ) No ( )

Breathlessness

5.Have you had an attack of shortness of breaththat came on during the day when being at rest at any time in the last ***12 months***? Yes ( ) No ( )

6.Have you had an attack of shortness of breath that came on following strenuous activity at any time in the ***last 12 months***? __ Yes ( ) No ( )

7.Have you ever been woken by an attack of shortness of breath in **the last 12 months**? ________ Yes ( ) No ( )

Asthma

8.Have you ever had asthma? ________ Yes ( ) No ( )

If yes, was the asthma diagnosed by a physician? ________ ( ) ( )

If yes, how old were you when you got the diagnosis by a physician? _______years age

If yes, how old were you when you had the **first** attack of asthma? _______years age

If yes, how old were you when you had **the most recent** attack of asthma? _______years age

9. Have you had any attack of asthma in the last **12 months**? _______ Yes ( ) No ( )

10. Are you currently using any asthma medication? (spray, inhalation powders, tablets)________ Yes ( ) No ( )

QUESTIONS ABOUT HEALTH AND CURRENT ALLERGIES

1. How many airway infections have you had

during the last 3 months? _______number

2. Have you used antibiotics (e.g. penicillin) against airway infections during the **last 12 months**?

**No, never**	**Yes, one time**	**Yes more than one time**
( )	( )	( )

3. Have you had any diseases you had to go to doctor? Yes ( ) No ( )

If yes, which diseases?__________________________

4. Are you a smoker? Yes ( ) No ( )

	Yes	No	Don't know
5. Do you have hypersensitivity/allergy to **cats**?	( )	( )	( )
6. Do you have hypersensitivity/allergy to **dogs**?	( )	( )	( )
7. Do you have hypersensitivity/allergy to **objects with moulds on**?	( )	( )	( )
8. Do you have hypersensitivity/allergy to **pollen**?	( )	( )	( )
9. Do you have food allergy?	( )	( )	( )

If yes, what is causing the allergy reactions?…………………………………………

ALLERGIC DISORDERS AMONG OTHER FAMILY MEMBERS

1. Number of older siblings, totally: ……..…

   Number of older siblings living at home now:…………………

2. Number of younger siblings, totally:..……

   Number of younger siblings living at home now:………………

3. Are there any allergic disorders in the family? Mark with an X in applicable places even if the symptoms has disappeared.

	Father	Mother	Siblings
Asthma	( )	( )	( )
Allergic nasal symptoms	( )	( )	( )
Eczema	( )	( )	( )

QUESTIONS ABOUT CHILDHOOD

Yes No

1. Were you breast feeded in your childhood?______________________ ( ) ( )

If yes, until you were_____________months old.

2. Did anyone in your family smoke from your birth until you were one year old?

	Yes	No
Father smoked	( )	( )
Mother smoked	( )	( )
Other family members smoked	( )	( )

	Yes, more than 3 years	Yes, 1-3 years	Yes, less than 1 year	No, never
3. Have you been attending any day care centres/nursery?	( )	( )	( )	( )

If yes, at what age were you first attending a day care centre/nursery?

Less than 1 year age( ) 1-2 years age( ) more than 2 years age( )

QUESTIONS ABOUT THE CURRENT HOME ENVIRONMENT

1. What type of building are you living in now? (answer by making a ring around one alternative)

Single family house **Detached house** **Apartment** **Farm** **Other type**

Yes No

2. Have you lived in the same building since your birth? ______________________( ) ( )

If no, which year did you move to the current dwelling? __________ (year)

3. Which year (approximately) was the house constructed? (year)

4. How many square meters living area has the dwelling?………(m^2)

5. Which material is used in the construction of your building? (answer by making a ring around one or more alternative)

Ston( ) Concrete( ) Brick( ) Wood( ) other material( )

Yes No

6. Have the interior of your dwelling been painted during the **last 12 months**? ( ) ( )

If yes, **when was it painted**?___________month____________year

Yes No

7. Have the floor in your dwelling been changed **last 12 months**? ___ ( ) ( )

8. Are there any **pets** in your dwelling?__________________________ ( ) ( )

If yes, what type of pet: _____________________________________

9. Have any of the following been identified in your dwelling during the **last 12 months**?

Yes No

*Water leakage or water damage indoors in walls, floor or ceiling*__________________________ ( ) ( )
Bubbles or yellow discoloration on plastic floor covering or black discoloration on parquet floor ( ) ( )
*Visible mould growth on indoors on walls, floor or ceilings*______________________________ ( ) ( )
*The smell of mould in one or more rooms (not the basement)*____________________________ _( ) ( )
*Any other smell(odours in the home, If yes describe the odour:*___________________________ ( ) ( )

Yes No

10. Is it common with dampness/condensation on the **lower part of the windows in winter**? ( ) ( )

11. Have any dampness problems/water damage occured in the dwelling during the **last 5 years**
( ) ( )

If yes, please give a description below of what was the cause of the dampness problem/water damage:

	Yes, daily	Yes, often 1-4 times/ week	Yes, sometimes 1-3 times/month	No, never
12. Is there any **tobacco smoking indoors** in the dwelling?	( )	( )	( )	( )

INFORMATION ABOUT CURRENT DIETARY HABITS

	Never	Rarely	Once a week	More than once per week	Almost daily
How often are you eating meat dishes?	( )	( )	( )	( )	( )
How often are you eating fish dishes?	( )	( )	( )	( )	( )
How often are you eating seafood dishes?	( )	( )	( )	( )	( )
How often are you eating fruit?	( )	( )	( )	( )	( )
How often are you eating vegetables(raw)?	( )	( )	( )	( )	( )
How often are you eating vegetables (cooked)?	( )	( )	( )	( )	( )
How often are you drinking milk?	( )	( )	( )	( )	( )
How often are you eating yoghurt, or other products from fermented milk?	( )	( )	( )	( )	( )
How often are you out eating fast food (hamburgers, pizza, hot dogs etc.)?	( )	( )	( )	( )	( )
How often are you drinking fruit juice?	( )	( )	( )	( )	( )
How often are you drinking soft drink?	( )	( )	( )	( )	( )

What types of fat/oils is used at cooking at home? (answer by making a ring around one or more alternative)

Butter( ) Margarine ( ) Olive oil ( ) Rape seed oil ( ) Other cooking oils ( )

CURRENT SYMPTOMS: HAVE YOU HAD ANY OF THE FOLLOWING SYMPTOMS DURING THE LAST <u>THREE MONTHS</u>:

	**Yes, daily**	**Yes, often 1-4 times/ week**	**Yes, sometimes 1-3 times/month**	**No, never**
1.Rashes on hands or forearms? ______________	( )	( )	( )	( )
2.Rashes on the face or throat? ______________	( )	( )	( )	( )
3.Eczema? If yes, where? ____________________	( )	( )	( )	( )
4.Itching in the face or on the throat? _________	( )	( )	( )	( )
5.Itches on hands or forearms? ______________	( )	( )	( )	( )
6.Eye irritation (redness, dryness, itch)?_______	( )	( )	( )	( )
7.Swollen eyelids? __________________________	( )	( )	( )	( )
8.Headache? _______________________________	( )	( )	( )	( )
9.Nausea? _________________________________	( )	( )	( )	( )
10.Runny nose/nasal catarrh? ________________	( )	( )	( )	( )
11 Nasal obstruction/blocked nose? __________	( )	( )	( )	( )
12.Throat dryness?__________________________	( )	( )	( )	( )

13.Sensation of catching a cold?______________ ( ) ( ) ( ) ( )

14.Sore throat? ____________________________ ( ) ( ) ( ) ( )

15.Irritative cought?________________________ ( ) ( ) ( ) ( )

16.Breathing difficulties?____________________ ( ) ( ) ( ) ( )

17. Feeeling tired and out of sort? ____________ ( ) ( ) ( ) ( )

Does any of these symptoms improve Yes No Do not know
*When you are away from **school**?* _________________________________ ( ) ( ) ( )

<u>If yes,</u> which symptoms? (write the number of the questions):_____

Does any of these symptoms improve Yes No Do not know
*When you are away from the **dwelling**?* ____________________________ ( ) ( ) ( )

<u>If yes,</u> which symptoms? (write the number of the questions):_____
